# Tissue remodeling macrophages morphologically dominate at the interface of polypropylene surgical meshes in the human abdomen

**DOI:** 10.1007/s10029-020-02315-2

**Published:** 2020-10-08

**Authors:** A. Dievernich, P. Achenbach, L. Davies, U. Klinge

**Affiliations:** 1 Department of General, Visceral and Transplant Surgery, RWTH Aachen University Hospital, Pauwelsstraße 30, 52074 Aachen, Germany; 2 Institute of Neuropathology, RWTH Aachen University Hospital, Aachen, Germany; 3 Division of Infection and Immunity, Cardiff University, Cardiff, UK

**Keywords:** Foreign body reaction, Macrophage, Collagen, Mesh, Fluorescence microscopy

## Abstract

**Background:**

Mesh implants are widely used to reinforce the abdominal wall, although the inevitable inflammatory foreign body reaction (FBR) at the interface leads to complications. Macrophages are suspected to regulate the subsequent scar formation, but it is still unclear whether adequate fibrous scar formation with collagen deposition depends mainly on the presence of M1 or M2 macrophages.

**Methods:**

This study investigated the FBR to seven human polypropylene meshes, which were removed after a median incorporation time of 1 year due to the primary complaint of recurrence. Using immunofluorescence, the FBR was examined in six regional zones with increasing distance from the mesh fibers up to 350 µm, based on the cell densities, macrophage M1 (CD86) and M2 (CD163, CD206) phenotypes, deposition of collagen-I and -III, and expression of matrix metalloproteinase-2 (MMP-2) and -8 as indicator of collagen degradation.

**Results:**

All mesh–tissue complexes demonstrated a decrease in cell density and macrophages with distance to the mesh fibers. Overall, about 60% of the macrophages presented an M2 phenotype, whereas only 6% an M1 phenotype. Over 70% of macrophages showed co-expression with collagen-I or -III and over 50% with MMP-2.

**Conclusions:**

The chronic FBR to polypropylene meshes is associated with an M2 macrophage response, which is accompanied by collagen deposition and MMP-2 expression. These findings challenge the idea that mainly M1 macrophages are related to inflammation and highlights that iatrogenic attempts to polarize these cells towards the M2 phenotype may not be a solution to ameliorate the long-term foreign body reaction.

**Electronic supplementary material:**

The online version of this article (10.1007/s10029-020-02315-2) contains supplementary material, which is available to authorized users.

## Introduction

Currently, textile mesh structures made of polypropylene are widely used to reinforce a hernia repair in the abdominal wall. The tissue reaction to the implants leads to the formation of a foreign body granuloma. Here, the mesh fibers are surrounded by dense layers of inflammatory cells with accompanying fibrotic encapsulation that shields the mesh from the surrounding tissue. After early recruitment of neutrophils, macrophages become the predominant cell mediator and are suspected to regulate the subsequent scar formation [1].

In 1992, Stein et al. demonstrated that IL-4 is an alternative activator for macrophages [2]. His findings later led to the separation of activated macrophages into two main populations, designated classically activated (M1) and alternatively activated (M2) [3]. This nomenclature is too simplistic and does not accurately describe the heterogeneity of macrophage phenotype in complex environments in situ; however, it remains useful for clinical assessment. Typically, in humans, ‘M1’ macrophages express the cell surface marker CD86 and produce high levels of proinflammatory cytokines. A prolonged or persistent M1 response can damage the tissue. In contrast, ‘M2’ macrophages encompass several different macrophage populations, which have overlapping marker expression and are relatively anti-inflammatory [4]. Though, two key markers are the mannose receptor CD206 and the scavenger receptor CD163. These cells produce lower amounts of proinflammatory cytokines and are considered to play a primary physiological role in promoting constructive healing and tissue remodeling [5]; hence, being termed by some as wound healing macrophages. However, it is recognized that a long-lasting strong ‘M2’ response can lead to excessive collagen formation, resulting in fibrosis [1, 6].

Experimental studies in animals with meshes have demonstrated that at least within 12 weeks after implantation, mainly M1 (CD86^+^) macrophages surrounded the mesh fibers and formed the foreign body granuloma [7, 8]. The predominance of these cells was confirmed by Nolfi et al. at human mesh explants from the pelvic floor, which were explanted because of inflammatory or fibrotic complications [9].

However, due to several conflicting studies, it still is not clear whether adequate fibrous scar formation with collagen deposition depends mainly on the presence of M1 or M2 macrophages [10]. In a mouse model, a shift from the M1 towards M2 phenotype led to a reduced fibrotic response [11], whereas in another mouse model the M2 phenotype was responsible for the development of fibrosis in the lung, likely by promoting myofibroblast differentiation of mesenchymal stem cells [12]. This highlights the need for further studies to determine the role of macrophages in both collagen deposition and degradation.

Therefore, we investigated whether M1 or M2 macrophages are related to local deposition of collagen-I and -III at seven polypropylene (PP) meshes, which were explanted from the abdominal wall due to recurrences. We examined the foreign body reaction to the meshes using cell densities, defined as cells per mm^2^, and the percentages of macrophage populations in six regional zones with increasing distance from the mesh up to 350 µm. In addition, we quantified the collagen deposition in each regional zone and the expression of two different matrix metalloproteinases: gelatinase A (MMP-2) and neutrophil collagenase (MMP-8) as indicators of collagen degradation.

## Materials and methods

We analyzed tissue sections of seven polypropylene (PP) monofilament meshes (used for the repair of abdominal wall hernias), which were explanted due to recurrences. After removal, samples were embedded in aqueous formalin solution to preserve the tissue before sectioning and further processing to FFPE blocks. Operative reports from the initial mesh surgery were reviewed, and the following meshes were recorded: 3 large pore Ultrapro®, 2 Ventralex® with a layer of PTFE, and 2 small pore plugs (Table 1). To optimize antibody dilutions and to check specificity of labeling, we used human spleen and tonsil tissues that did not display gross pathology as control tissues (Online Resource 1).

**Table 1:** Information on the patients whose mesh samples were examined in this study

Explant no	Mesh type	Incorporation time (years)	Gender	Age
#1	Ultrapro®	0.6	Female	52
#2	Ultrapro®	0.9	Female	34
#3	Ultrapro®	1.3	Female	50
#4	Ventralex®	1.0	Male	47
#5	Ventralex®	6.0	Female	57
#6	Plug	2.2	Male	69
#7	Plug	4.0	Male	51

**Fig. 1:**
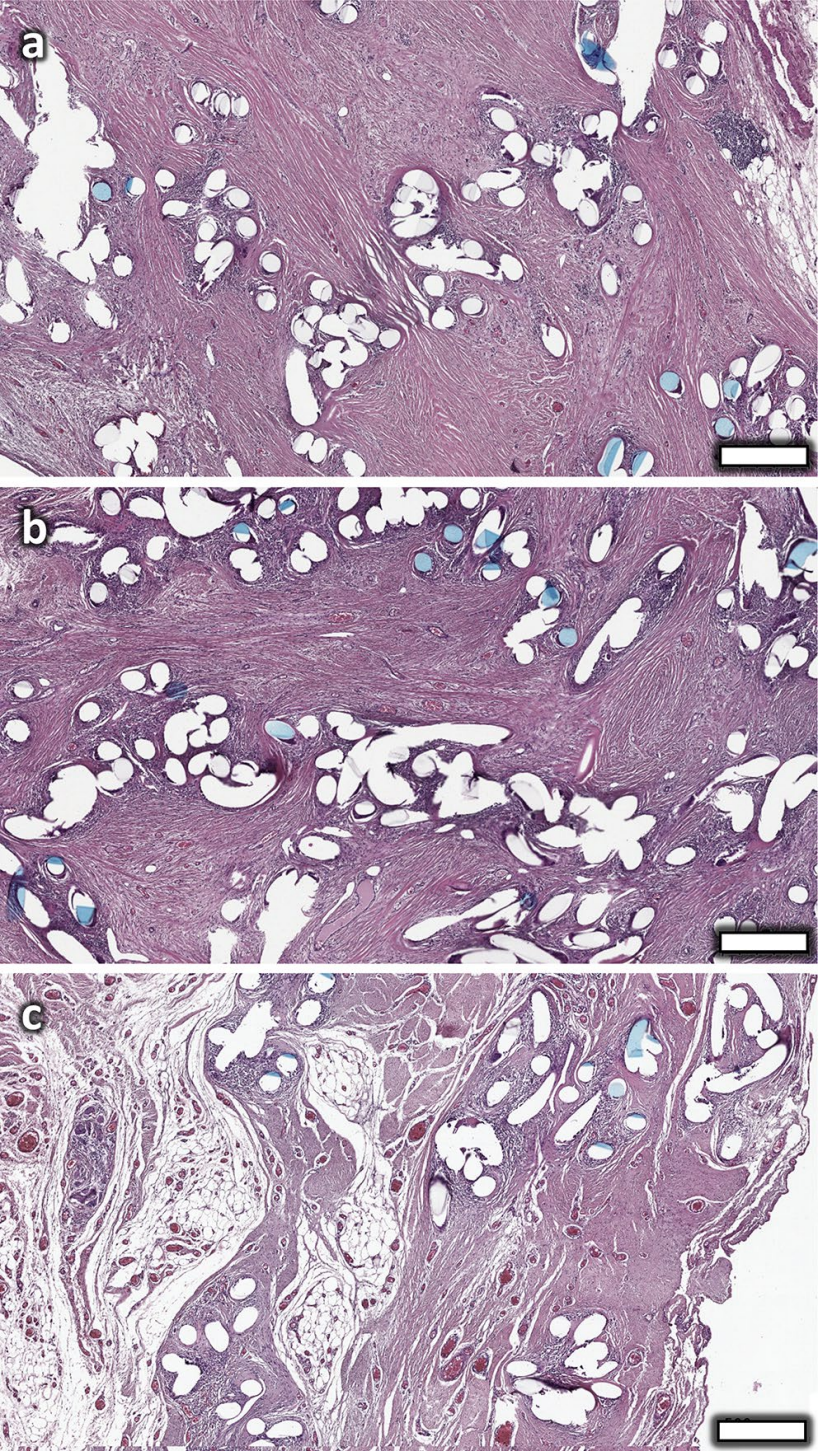
HE stainings of three representative PP mesh samples with fibrosis. The mesh fibers are encapsulated by abundant fibrous tissue that bridges between adjacent mesh fiber bundles. Scale bar = 500 µm. Images of explants #6 (**a**), #7 (**b**), #2 (**c**)

Prior to immunofluorescence staining, mesh samples were checked for the presence of characteristic cells and morphology by hematoxylin and eosin (Fig. 1). All specimens showed a typical, highly localized foreign body reaction around the mesh fibers with an inner infiltrate of predominantly mononuclear cells and an outer fibrotic capsule. Multinucleated giant cells were observed at the surface of some mesh fibers in all samples.

### Immunofluorescent staining

#### General

All steps were performed at room temperature. Serial 2 µm sections of each specimen were double labeled with monoclonal antibodies (Table 2). The order of fluorophores was always kept constant. For the first marker we always used the pan-macrophage marker CD68 labeled with fluorescein isothiocyanate (FITC). The second marker was always labeled with cyanine-5 (Cy5). The list of second markers includes CD86 (M1), CD163 and CD206 (both M2), collagen-I, collagen-III, MMP-2, and MMP-8. All antibodies were diluted with Antibody Diluent (with Background Reducing Components, Dako, Germany). Secondary antibodies were applied with ImmPRESS™ HRP (Peroxidase) Polymer Detection Kit (Vector Laboratories, US). TSA reagents were diluted with 1× Plus Amplification Diluent (PerkinElmer/Akoya Biosciences, US).

**Table 2:** List of monoclonal antibodies sorted alphabetically

Antibody	Clone	Dilution	Incubation time	Manufacturer
CD68	KP1	1:6000	30 min	Dako
CD163	5C6 FAT	1:800	Over night	BMA Biomedicals
CD206	15–2	1:200	30 min	Acris
Collagen-I	5D8-G9	1:125	Over night	ThermoFisher
Collagen-III	FH-7A	1:200	30 min	ThermoFisher
MMP-2	CA-4001 or CA719E3C	1:300	Over night	ThermoFisher
MMP-8	EP1232Y	1:300	Over night	abcam

#### Protocol

Tissue sections were deparaffinized with xylene, rehydrated through graded alcohol and Milli-Q, before incubation in 3.5% formalin for 10 min. Sections were then placed in a cuvette filled with Milli-Q and pH6 citrate buffer (1:10) and treated for 10 min at 110 °C in a Decloaking Chamber™ (Biocare Medical, US) for antigen retrieval. Afterwards, sections were washed with Milli-Q and TBST Tris (Buffered Saline with Tween 20, Dako) and cooled. Nonspecific binding was blocked by incubation with antibody diluent for 10 min.

These steps were followed by incubation with the primary antibody of the first marker. After incubation, sections were rinsed in TBST Tris and incubated with the secondary antibody for 20 min, before applying FITC-staining with the Opal™ 520 Reagent Pack (1:100, PerkinElmer/Akoya Biosciences) for 10 min. Sections were then washed with TBST Tris and placed in a cuvette filled with AR6 Buffer (PerkinElmer/Akoya Biosciences) and Milli-Q (1:10). For antibody stripping, the cuvette was microwave treated for 3 min at 385 W reaching a maximal temperature of 92 °C and 15 min at 120 W reaching a maximal temperature of 90 °C, before being cooled with cold water. Sections were removed and rinsed with Milli-Q, before incubation with TBST Tris overnight.

The second marker was applied on the following day. After applying the primary and secondary antibodies of the second marker, sections were Cy5-stained with the TSATM-Plus Cyanine 5 System (1:50, PerkinElmer/Akoya Biosciences). Sections were then rinsed in Milli-Q and finally counterstained and cover-slipped with VECTRASHIELD® HardSet™ Antifade Mounting Medium with DAPI (Vector Laboratories).

### Analysis of fluorescence images/stainings

**Fig. 2:**
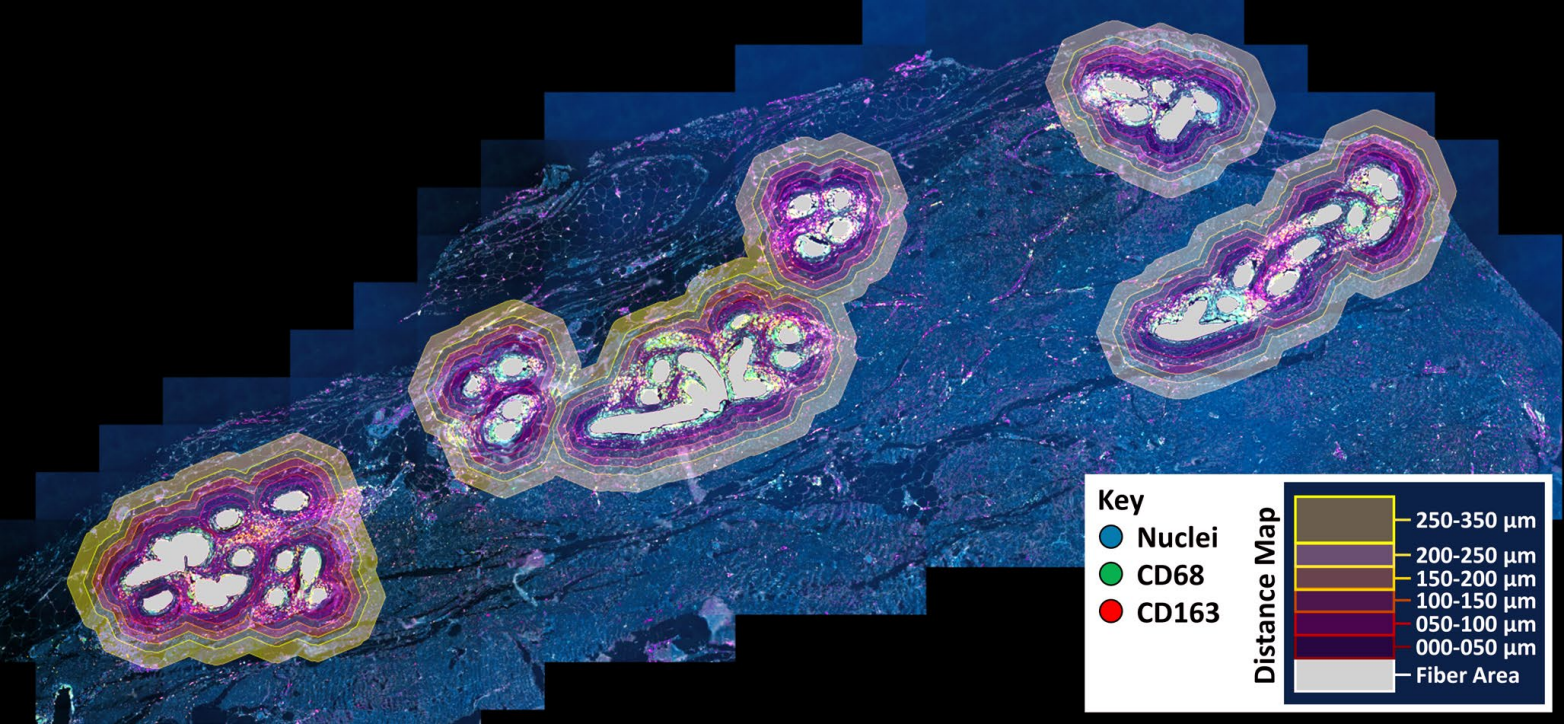
Immunofluorescent labeling with distance map algorithm for spatial analysis of the host reaction to mesh implants. Tissue section of a human mesh explant with selected fiber areas in gray and the automatically generated Euclidean distance map. The distance map consists of six regional zones covering a total distance of 350 µm from the mesh fibers. Image of explant #3

**Fig. 3:**
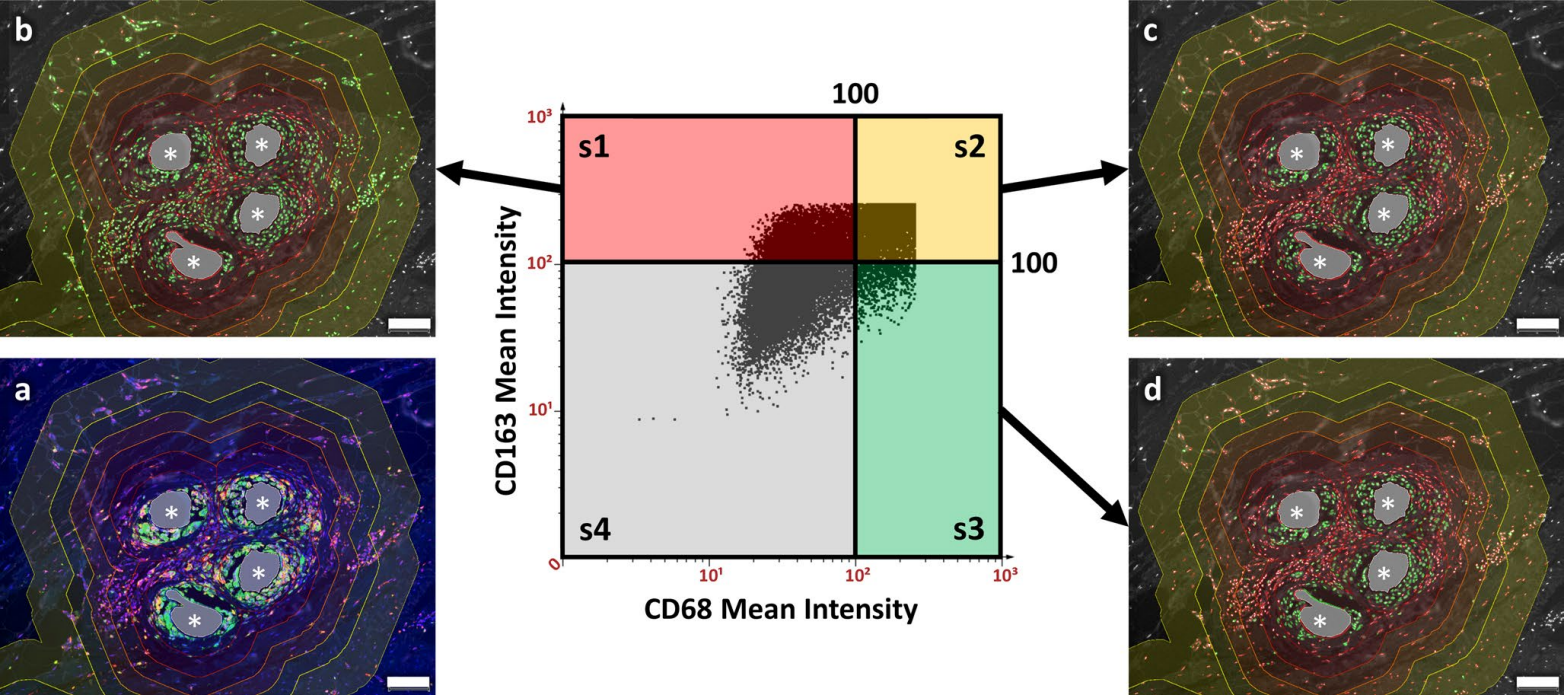
Backward gating of macrophages participating in the foreign body reaction. **a** Immunofluorescent labeling of a human mesh explant for CD68 (green), CD163 (red), and nuclei with DAPI (blue). (s1–s4) Scatter plot of the mean cell intensities with CD68 on the *x* axis and CD163 on the *y* axis. Cells with a mean staining intensity > 100 are considered to be “positive” and shown in green in the backward gating images (**b**–**d**). Backward gating of cells was done on DAPI grayscale images and compared with color images to verify detection. **b** Backward gating of CD163^+^ cells (= s1 and s2). **c** Backward gating of CD68^+^CD163^+^ M2 macrophages (= s2). **d** Backward gating of CD68^+^ macrophages (= s2 and s3). All images are superimposed with the regional zones of the Euclidean distance map that range from 0 to 50 µm (dark red) to 250–350 µm (bright yellow) in 50 µm steps. Mesh fibers are marked with asterisks, scale bars = 100 µm. Images of explant #3 (color figure online)

**Fig. 4:**
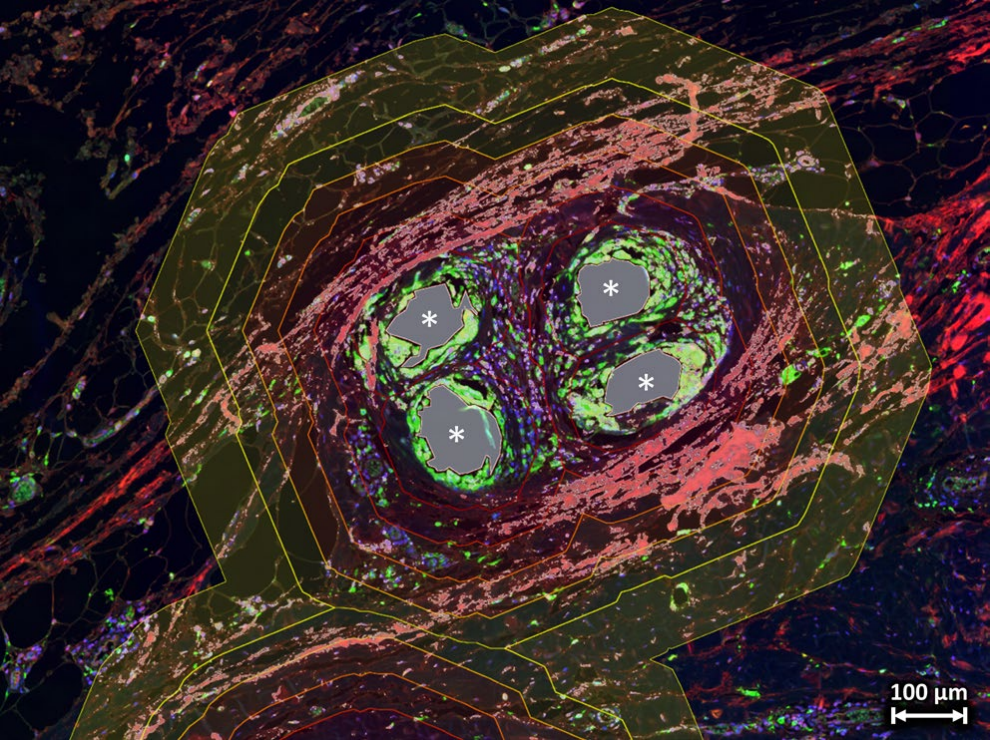
Collagen detection in the vicinity of mesh fibers. Double-stained tissue section of a human mesh explant labeled for CD68 (green) and collagen-III (red) with superimposed fiber areas in gray and the regional zones of the Euclidean distance map (0–50 µm in dark red to 250–350 µm in bright yellow). Detected collagen (pixel intensity > 100) within the zones is highlighted with a gray shade and the number of pixels was counted to determine the total collagen area in each regional zone. Mesh fibers are marked with asterisks. Image of explant #3 (color figure online)

Fluorescence imaging was performed with an Axio Imager 2 epifluorescence microscope (20x, Zeiss, Germany) and the TissueFAXS PLUS system (TissueGnostics, Austria). Images were processed and quantitatively analyzed with StrataQuest Analysis Software (v6, TissueGnostics). On average, 41 (range: 10–130) individual mesh fibers were manually outlined in each mesh sample and processed using the Euclidean distance to establish Euclidean distance maps with six regional zones (zone 1: 0–50 µm, zone 2: 50–100 µm, zone 3: 100–150 µm, zone 4: 150–200 µm, zone 5: 200–250 µm, zone 6: 250–350 µm) from the mesh fibers (Fig. 2). The Euclidean distance is a method commonly used in image processing that uses the Pythagorean formula to measure the distance between two pixels in a straight line. The detection of cells and positive marker signals was done as described previously [13]. Briefly, optimized DAPI images were used to detect and segment nuclei whose areas were used to measure the mean staining intensities for FITC- and Cy5-shades of the respective markers. Cells whose mean staining intensity was above 100 were considered “positive” and detection was verified with backward gating (Fig. 3). In addition, for collagen-I and -III the positive signal area (pixels with an intensity > 100) was detected for each regional zone (Fig. 4). We recorded the total area of each zone, the total area of collagen, the total number of nuclei, as well as the percentages of FITC^+^ (CD68^+^), Cy5^+^ (second marker^+^), and double positive, FITC^+^Cy5^+^ (CD68^+^ and second marker^+^), cells in each zone.

In addition, controls without primary antibody and controls with isotype antibodies were performed. The omission of the primary antibodies and substitution of the primary antibodies with the isotype antibodies at the same final concentrations resulted in lack of immunostaining.

### Statistical analysis

Calculations were done with MATLAB® 9.1 and Image Processing Toolbox 9.5 (The MathWorks, US). Statistical analysis was performed with Statistical Package for Social Sciences software (SPSS® v23, IBM, US).

## Results

### Distribution of cell densities and macrophages in relation to the distance from the mesh fibers

**Fig. 5:**
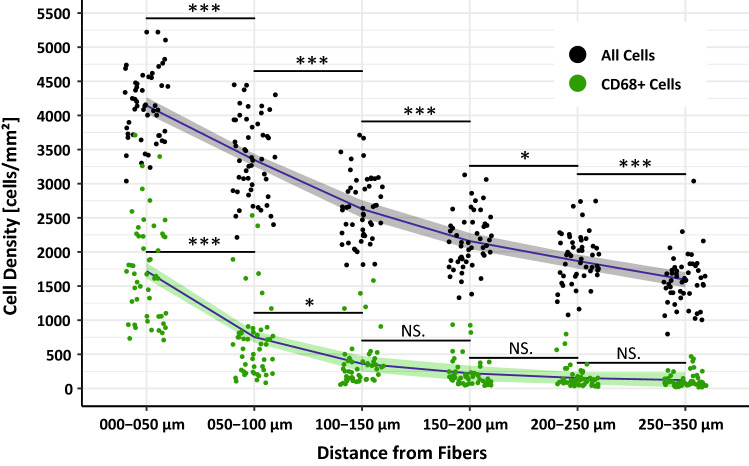
Spatial distribution of cell densities and macrophages at the mesh–tissue interface. Dots correspond to the mean cell densities (cells per mm^2^) of all cells and macrophages (CD68^+^) in each regional zone, e.g., 0–50 µm. Statistical significance between neighboring zones were determined with Welch-ANOVA followed by Dunnett’s T3 post hoc test (***p < 0.001, **p < 0.01, *p < 0.05, NS. = not significant). The gray- and green-shaded areas mark the 95% confidence interval of the respective mean values for macrophages and all cells (blue lines). *N* = 7 meshes each 7–9 stains (color figure online)

Mesh–tissue complexes exhibited a typical highly localized foreign body reaction with inner inflammatory cell infiltrates and outer fibrotic collagen-containing capsules (Fig. 4). To determine the percentage of macrophages (as function of total cells) in each zone, we first studied cell densities, defined as cells per mm^2^. The cell density decreased with increasing distance from the mesh fibers from 4133 cells/mm^2^ in zone1 (0–50 µm) to 1583 cells/mm^2^ in zone6 (250–350 µm). The number of CD68^+^ macrophages showed a similar decline, although the percentages decreased even more rapidly, from 41.7 to 7.5%, respectively (Fig. 5; Table 3).

**Table 3:** Spatial analysis of cell densities, macrophages, and M2/M1 ratios

Parameter (*n* = 7 PP meshes)	Distance from Fibers					
	Zone1 000–050 µm	Zone2 050–100 µm	Zone3 100–150 µm	Zone4 150–200 µm	Zone5 200–250 µm	Zone6 250–350 µm
Cell density (cells/mm^2^)	4133.8 (40.2)	3387.1 (82.9)	2628.3 (66.9)	2158.4 (56.5)	1904.8 (52.0)	1583.6 (52.0)
% CD68^+^ cells	41.7 (2.3)	19.7 (2.1)	13.6 (1.6)	10.3 (1.3)	8.5 (1.1)	7.5 (0.9)
% CD86^+^ cells	2.9 (1.2)	2.2 (0.8)	2.2 (0.7)	2.6 (0.9)	2.6 (0.9)	2.6 (0.9)
% CD68^+^CD86^+^ cells (= M1)	1.7 (0.6)	0.4 (0.1)	0.3 (0.1)	0.4 (0.1)	0.2 (0.1)	0.3 (0.1)
% CD68^+^CD86^+^/CD68^+^	6.5 (2.6)	4.1 (1.7)	3.8 (1.4)	6.4 (2.0)	4.5 (1.4)	8.8 (3.8)
% CD163^+^ cells	41.1 (6.1)	33.6 (5.5)	29.8 (5.1)	27.5 (4.8)	28.7 (4.8)	28.1 (5.0)
% CD68^+^CD163^+^ cells (= M2 [1])	23.1 (3.6)	10.2 (1.9)	6.8 (1.2)	4.7 (0.8)	4.2 (0.7)	3.2 (0.4)
% CD68^+^CD163^+^/CD68^+^	58.6 (9.2)	65.2 (7.4)	66.5 (6.1)	66.8 (6.3)	71.3 (6.4)	73.3 (7.2)
% CD206^+^ cells	22.3 (1.9)	10.7 (2.3)	8.5 (1.8)	7.5 (1.3)	6.9 (1.2)	6.1 (1.2)
% CD68^+^CD206^+^ cells (= M2 [2])	18.3 (1.3)	6.5 (1.6)	4.5 (1.1)	3.4 (0.8)	3.1 (0.7)	2.6 (0.6)
% CD68^+^CD206^+^/CD68^+^	57.2 (3.8)	52.9 (4.2)	57.6 (4.9)	59.5 (5.8)	63.8 (6.7)	60.6 (7.3)
M2[1]/M1 ratio	42.4 (20.1)	45.7 (17.3)	67.3 (31.0)	20.5 (7.3)	29.1 (8.4)	12.5 (4.4)
M2[2]/M1 ratio	57.9 (34.7)	38.3 (15.8)	70.8 (46.4)	24.8 (14.6)	23.5 (8.1)	11.0 (5.1)

The cell density is defined as the average number of cells per mm^2^. Percentages of single-positive (e.g., CD68^+^) and double-positive (e.g., CD68^+^CD86^+^) cells in relation to all cells and the proportions of double-positive cells with respect to the total number of macrophages (e.g., CD68^+^CD163^+^/CD68^+^) in each regional zone. M2/M1 ratios are given for both M2 marker combinations with the designations M2[1] and M2[2]. The data are presented as mean (SE)

### Spatial analysis of macrophage phenotypes

**Fig. 6:**
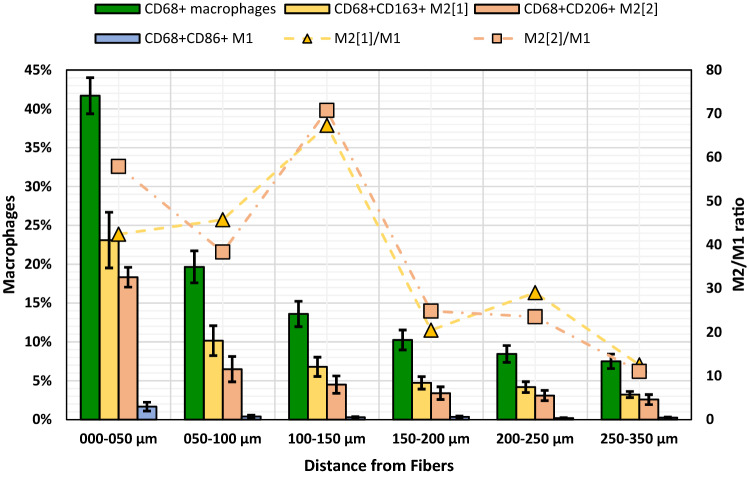
Characterization of the macrophage response to human polypropylene mesh explants. The green bars represent the mean percentages of macrophages (CD68^+^), yellow and orange of M2 macrophages (e.g., CD68^+^CD163^+^), and blue of M1 macrophages (CD68^+^CD86^+^) in each regional zone. The respective M2/M1 ratios are indicated with the yellow triangles (CD68^+^CD163^+^/CD68^+^CD86^+^) and orange squares (CD68^+^CD206^+^/CD68^+^CD86^+^). The percentage scale for the macrophages is on the left and for the M2/M1 ratios on the right. Whiskers mark the SEs (*n* = 7 meshes) (color figure online)

Separate analysis for M1 (CD68^+^CD86^+^) and M2 phenotypes (CD68^+^CD163^+^ and CD68^+^CD206^+^) revealed only few M1 macrophages with the highest percentage of 1.7% in zone1. In contrast, M2 macrophages were seen more frequently (Fig. 6; Table 3). As with the M1 macrophages, we observed the highest percentage of M2 macrophages in zone1 with 18.3% and 23.1% for CD68^+^CD206^+^ and CD68^+^CD163^+^, respectively. The relative proportions of M2 macrophages in relation to the total number of macrophages (e.g., CD68^+^CD163^+^/CD68^+^) remained constant in each zone. The overall M2/M1 ratios were 28.8 and 39.6 for CD68^+^CD163^+^ and CD68^+^CD206^+^, respectively, with similar zonal profiles of the ratios for both M2 marker combinations (Fig. 6). Collectively, these data demonstrate that these PP meshes presented a localized sustained M2 response.

### Collagen deposition and co-localization with macrophages

**Fig. 7:**
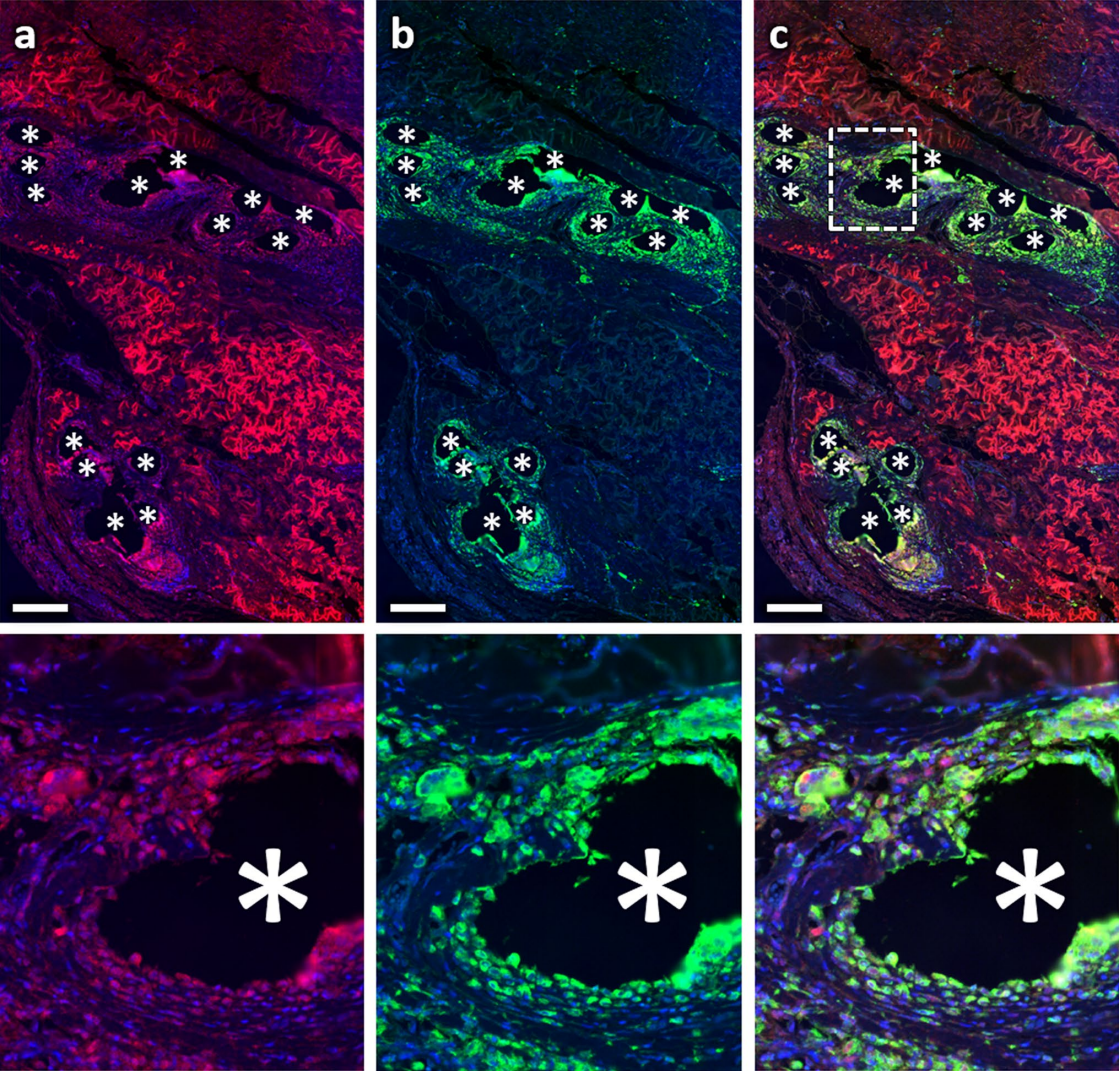
Immunofluorescence labeling of macrophages (CD68) and collagen-I. **a** Labeling of collagen-I (Cy5, red), **b** CD68 (FITC, green), and **c** overlay. Nuclei are labeled with DAPI (blue). Images corresponding to the dashed rectangular ROI are given below, respectively. Locations of mesh fibers are marked with asterisks, scale bar = 200 µm. Images of explant #3 (color figure online)

**Fig. 8:**
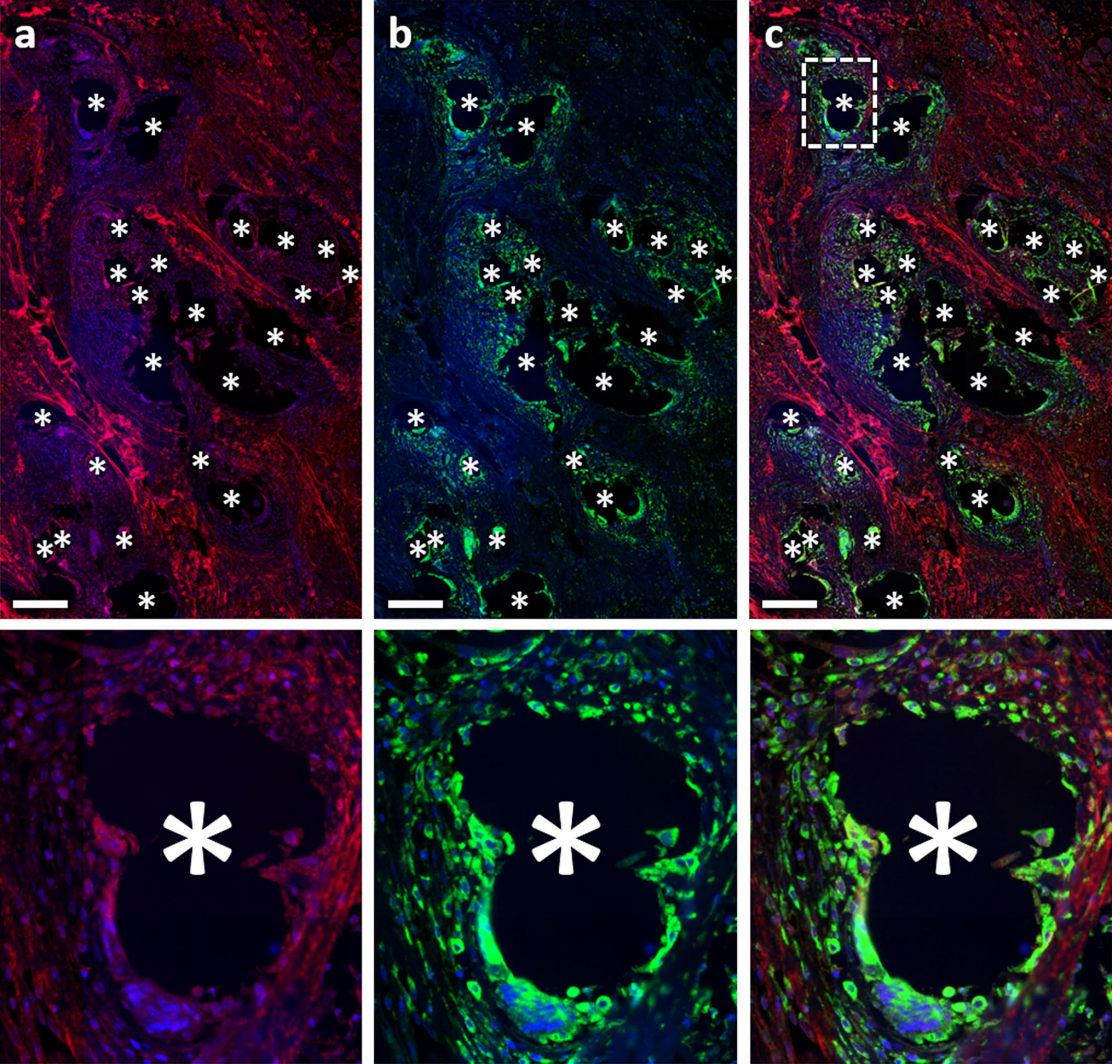
Immunofluorescence labeling of macrophages (CD68) and collagen-III. **a** Labeling of collagen-III (Cy5, red), **b** CD68 (FITC, green), and **c** overlay. Nuclei are labeled with DAPI (blue). Images corresponding to the dashed rectangular ROI are given below, respectively. Locations of mesh fibers are marked with asterisks, scale bar = 200 µm. Images of explant #7 (color figure online)

**Fig. 9:**
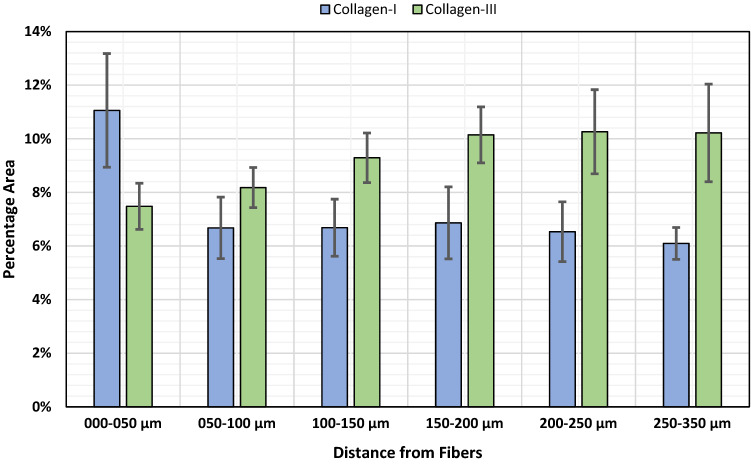
Spatial analysis of collagen-I and -III deposition. Bars represent the average percentage areas of collagen-I (blue) and collagen-III (green) in relation to the total area of the corresponding regional zones. Whiskers mark the SEs. No statistically significant differences were found between any zones (*n* = 7 meshes) (color figure online)

**Fig. 10:**
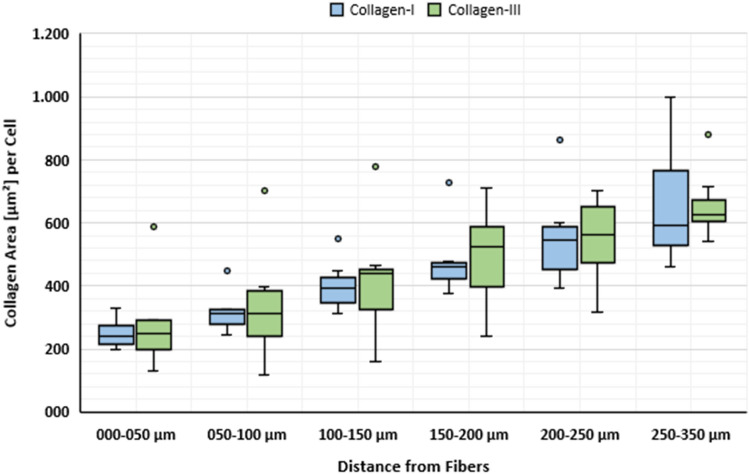
Regional distribution of collagen-I and -III per cell. Box plot with Tukey whiskers represents the average collagen-I (blue) and -III (green) area per cell in each regional zone (*n* = 7 meshes) (color figure online)

To investigate collagen deposition, we determined the area of collagen-I and -III as well as its co-localization with cells and macrophages (Figs. 7, 8) in each regional zone. For collagen-I, the percentage area (based on the total area) was highest in zone1 with about 11%, while it was relatively constant in the other zones with about 6.5%. In contrast, the percentage area of collagen-III increased steadily from about 7.5% in zone1 to about 10.2% in zone4 (150–200 µm) and remained constant in the two most distant zones (Table 4; Fig. 9). By relating the collagen areas in each regional zone to the respective number of all cells, we found that the average collagen area per cell for both collagens increased with increasing distance from the mesh fibers (Fig. 10). Furthermore, both collagens showed considerable co-localization with CD68^+^ cells, highest for collagen-I in zone1 (28.1%), and for collagen-III in zone5 (200–250 µm) and zone6 (250–350 µm). Overall, about 50% of macrophages co-localized with collagen-I and about 28% with collagen-III.

**Table 4:** Spatial distributions of collagen-I and collagen-III as well as co-localization with cells and macrophages

Parameter (*n* = 7 PP meshes)	Distance from Fibers					
	Zone1 000–050 µm	Zone2 050–100 µm	Zone3 100–150 µm	Zone4 150–200 µm	Zone5 200–250 µm	Zone6 250–350 µm
% Col-I^+^ area	11.1 (2.1)	6.7 (1.2)	6.7 (1.1)	6.9 (1.3)	6.5 (1.1)	6.1 (0.6)
% Col-III^+^ area	7.5 (0.9)	8.2 (0.8)	9.3 (0.9)	10.2 (1.0)	10.3 (1.6)	10.2 (1.8)
Col-I^+^/Col-III^+^ area ratio	1.4 (0.2)	0.9 (0.2)	0.9 (0.3)	0.8 (0.3)	0.8 (0.3)	0.7 (0.1)
% Col-I^+^ cells	28.1 (5.8)	16.7 (4.0)	14.9 (2.6)	14.8 (2.3)	15.9 (1.7)	17.8 (1.3)
% CD68^+^Col-I^+^ cells	19.1 (4.3)	6.7 (2.3)	4.2 (1.3)	2.7 (0.8)	2.3 (0.8)	2.3 (0.5)
% CD68^+^Col-I^+^/CD68^+^	52.2 (8.4)	46.3 (8.6)	46.7 (8.8)	42.3 (9.1)	40.4 (7.5)	44.4 (7.2)
% Col-III^+^ cells	17.7 (2.7)	15.1 (2.2)	16.4 (1.9)	18.6 (1.9)	20.2 (2.8)	22.5 (2.5)
% CD68^+^Col-III^+^ cells	10.2 (2.3)	3.0 (0.8)	2.3 (0.5)	1.6 (0.2)	1.2 (0.2)	1.5 (0.2)
% CD68^+^Col-III^+^/CD68^+^	29.3 (5.4)	22.5 (3.2)	28.4 (2.7)	30.7 (1.1)	29.1 (2.2)	36.5 (2.3)

Percentage areas of collagen-I and -III (e.g., Col-I^+^ area) as function of the total area of each regional zone with corresponding collagen ratios. Percentages of cells (e.g., Col-I^+^ cells) and macrophages (e.g., CD68^+^Col-I^+^ cells) co-localizing with collagen-I and -III, and the proportions of co-localizing macrophages relative to the total number of macrophages (e.g., CD68^+^Col-I^+^/CD68^+^) in each regional zone. The data are presented as mean (SE)

### Distribution of MMP-2 and MMP-8 and co-expression with macrophages

**Fig. 11:**
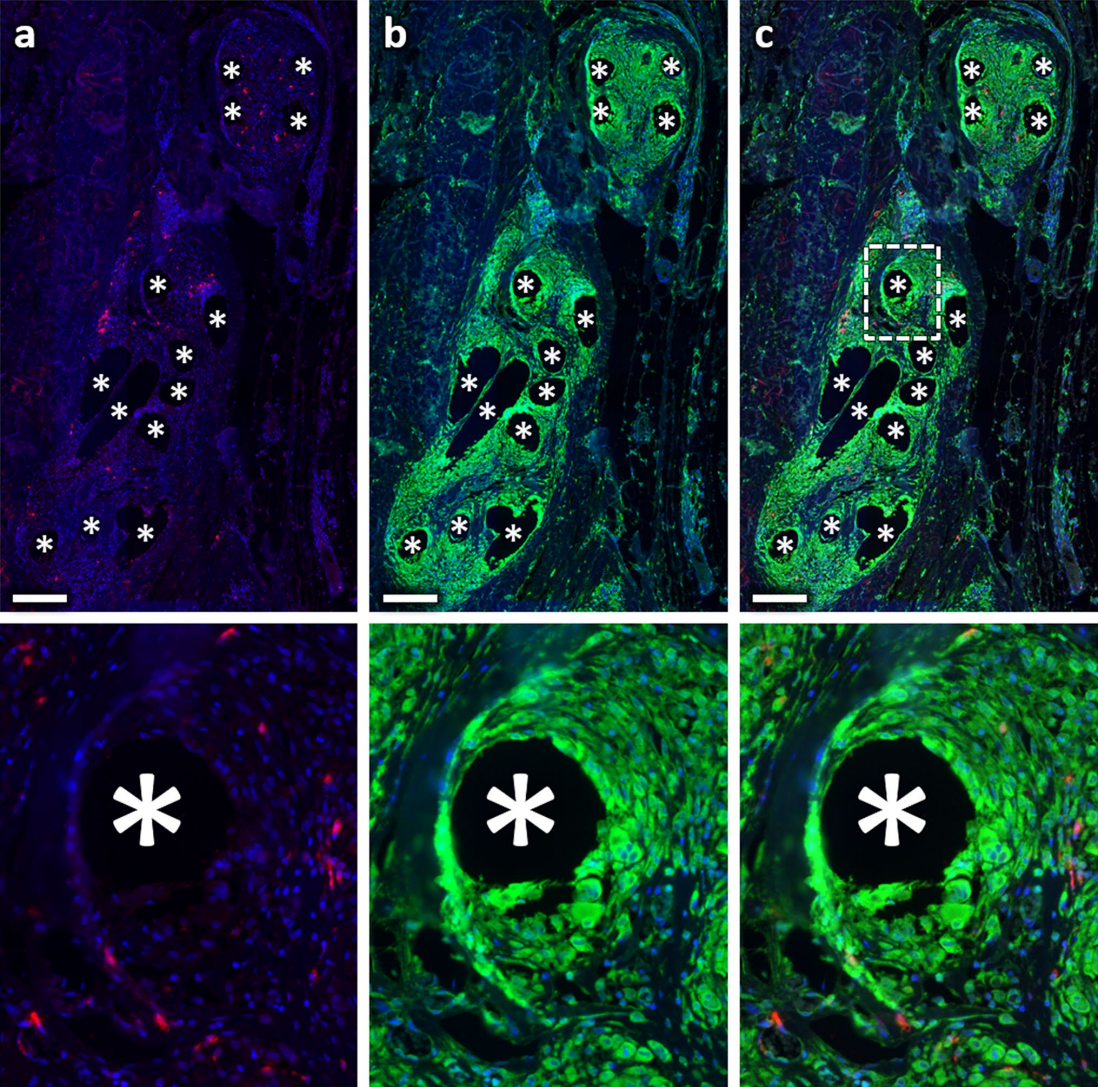
Immunofluorescence labeling of macrophages (CD68) and MMP-8. **a** Labeling of MMP-8 (Cy5, red), **b** CD68 (FITC, green), and **c** overlay. Nuclei are labeled with DAPI (blue). Images corresponding to the dashed rectangular ROI are given below, respectively. Locations of mesh fibers are marked with asterisks, scale bar = 200 µm. Images from explant #3 (color figure online)

**Fig. 12:**
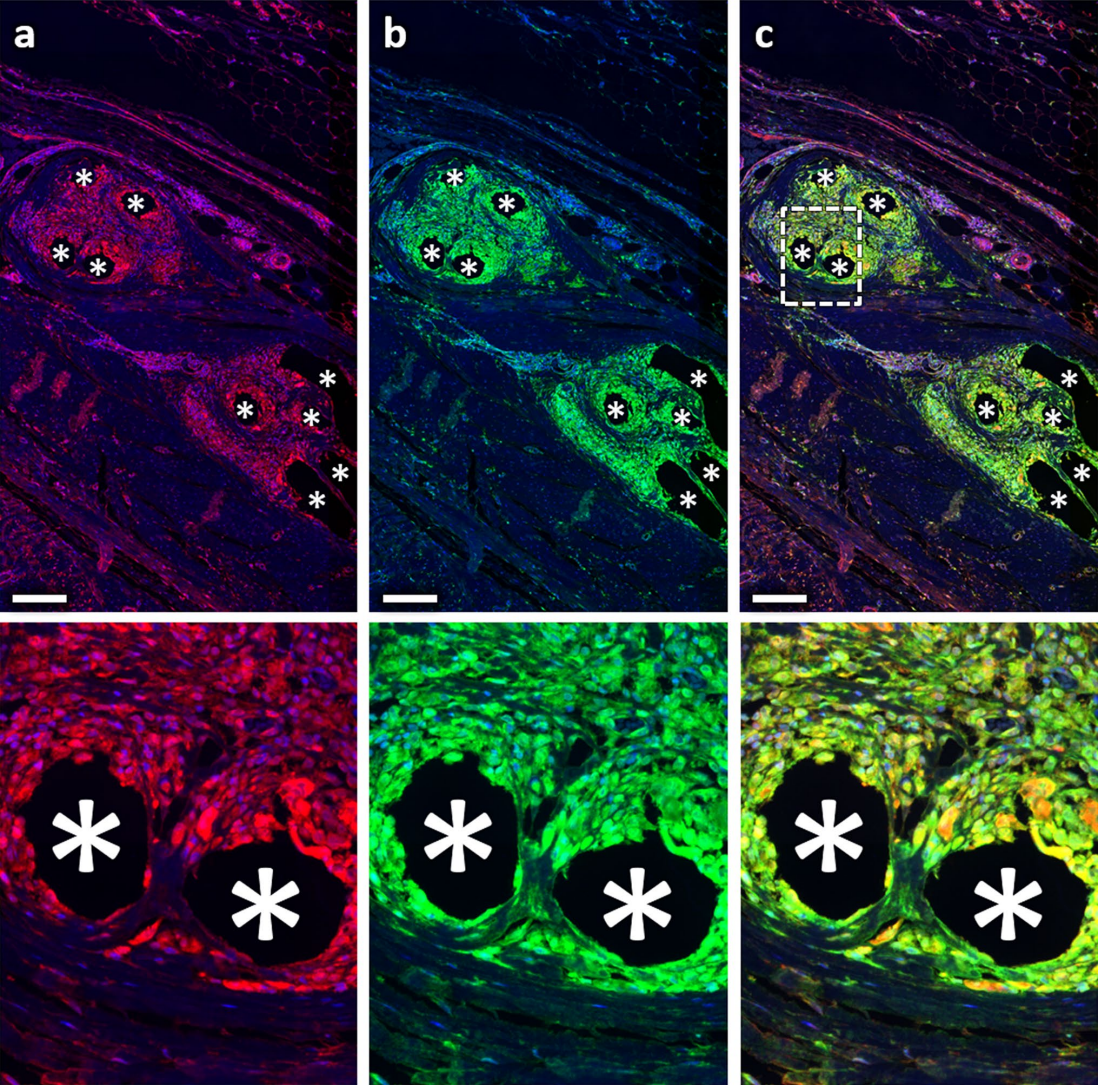
Immunofluorescence labeling of macrophages (CD68) and MMP-2. **a** Labeling of MMP-2 (Cy5, red), **b** CD68 (FITC, green), and **c** overlay. Nuclei are labeled with DAPI (blue). Images corresponding to the dashed rectangular ROI are given below, respectively. Locations of mesh fibers are marked with asterisks, scale bar = 200 µm. Images from explant #3 (color figure online)

Spatial analysis of matrix metalloproteinases revealed only little MMP-8 expression (Fig. 11) but considerable expression of MMP-2 (Fig. 12), almost equally distributed in all zones. Overall, about 55% of the macrophages co-expressed MMP-2 in each zone (Table 5).

**Table 5:** Spatial distribution of cells and macrophages co-expressing MMP-2 and MMP-8

Parameter (*n* = 7 PP meshes)	Distance from Fibers					
	Zone1 000–050 µm	Zone2 050–100 µm	Zone3 100–150 µm	Zone4 150–200 µm	Zone5 200–250 µm	Zone6 250–350 µm
% MMP-2^+^ cells	42.8 (5.9)	31.7 (5.9)	30.8 (6.1)	31.8 (5.7)	32.5 (6.2)	32.9 (5.8)
% CD68^+^MMP-2^+^ cells	38.6 (4.7)	21.2 (3.8)	16.3 (3.4)	13.6 (2.7)	12.0 (2.7)	10.5 (2.6)
% CD68^+^MMP-2^+^/CD68^+^	58.1 (7.9)	51.6 (8.3)	52.8 (8.7)	55.2 (8.8)	56.2 (9.1)	56.9 (8.8)
% MMP-8^+^ cells	2.5 (1.0)	2.2 (0.6)	2.2 (0.8)	2.9 (1.4)	2.8 (1.4)	2.6 (1.1)
% CD68^+^MMP-8^+^ cells	1.7 (0.8)	0.8 (0.3)	0.7 (0.3)	0.8 (0.5)	0.5 (0.2)	0.6 (0.4)
% CD68^+^MMP-8^+^/CD68^+^	3.1 (1.4)	2.5 (0.8)	3.0 (1.0)	3.7 (1.9)	4.3 (1.7)	5.4 (3.4)

Percentages of cells (e.g., MMP2^+^ cells) and macrophages (e.g., CD68^+^MMP-2^+^ cells) co-expressing MMPs with respect to all cells, and the proportions of co-expressing macrophages with respect to the total number of macrophages (e.g., CD68^+^MMP-2^+^/CD68^+^) in each regional zone. The data are presented as mean (SE)

## Discussion

The examination of the spatial morphology of the macrophages at the foreign body granuloma around meshes by combining a custom Euclidean distance map algorithm with immunofluorescence staining of entire tissue sections of polypropylene (PP) mesh explants demonstrated that cell density and macrophage ratio decreased with distance to the PP fibers, and that these were predominantly M2 macrophages. In addition, more than 70% of the macrophages showed co-expression with collagen-I or -III and more than 50% with MMP-2, which demonstrates these cells are important participants in mesh-tissue remodeling.

When examined, the cell densities and the percentages of macrophages decreased with increasing distance from the mesh fibers. Interestingly, the proportion of M2 macrophages in relation to the total number of macrophages was not significantly different between regional zones, highlighting the consistent M2 environment. The highest percentages of both M1 and M2 macrophages in situ were observed within the first 50 µm from the mesh surface, which is in agreement with other studies on PP meshes [8, 11, 14]. On the contrary to our findings, several animal studies on PP meshes have shown mainly M1 macrophages to surround the mesh fibers [7, 8, 11]. The predominance of M1 macrophages was confirmed by Nolfi et al. at human PP mesh explants from the pelvic floor, which were explanted due to mesh exposure and chronic pain [9]. Any quantification strongly depends on marker, cutoff, the distance to the mesh fibers, and the location of the investigated region, however the difference we record here is striking (‘M1’ ≈ 6% vs ‘M2’ ≈ 60%). The mesh placement at the pelvic floor may favor proinflammatory morphology of macrophages, whereas locations within the abdominal wall tends to show less inflammation [9, 15]. In addition, the medical reason for mesh removal may be decisive for macrophage morphology. Our explants represent the chronic foreign body reaction, but they have not been removed because of acute clinically apparent inflammation or erosion. Finally, M1/M2 morphology in humans may differ to the results in animals, especially considering many of these markers are not shared and that meshes are not adapted to the physiology of animals but rather to that of humans.

The M2 response observed in this study was accompanied by abundant collagen deposition, leading to fibrosis. This is in agreement with several studies on fibrotic pathogenesis like pulmonary fibrosis or hepatic fibrosis [12, 16–19]. Most collagen-I was found directly at the interface to the meshes where the cell density and percentage of M2 macrophages was highest, while in the more distant zones there was more collagen-III, but significantly less M2 macrophages. These observations are consistent with the knowledge that profibrotic M2 macrophages produce various mediators that directly activate fibroblasts known to be mainly located in the outer fibrotic capsule, which in turn control ECM deposition [19]. The observed co-localization of macrophages with collagen-I, especially directly at the mesh–tissue interface (> 50%), could indicate that M2 macrophages are responsible for collagen-I degradation via a mannose receptor-mediated (CD206) pathway after MMP cleavage, as discovered by Madsen et al. [20]. Alternatively or additionally, macrophages may be involved in the synthesis of collagen-I, since the synthesis of other collagen types has been demonstrated in animals and humans [21, 22].

Profibrotic M2 macrophages are also known to produce their own MMPs, which regulate inflammatory cell recruitment and ECM turnover [19]. MMP-8 was rarely observed, whereas abundant MMP-2 expression was present in all regional zones, which is consistent with previous studies that reported an up-regulated MMP-2 protein synthesis and enzymatic activity following mesh implantation [9, 23, 24]. About half of the macrophages were found to produce MMP-2. MMP-2 is a gelatinase that degrades collagens, its production by macrophages here may be an attempt to remodel the foreign body. However, the side effects may cause instability of the granuloma, as observed in atherosclerotic plaque rupture [25], and likely inhibit normal scar formation and maintain persistent cell turnover. Future studies will have to verify functionality of MMPs, e.g., by ELISA, zymography techniques, or RT-PCR gene expression, although the overall proteinase activity is altered by tissue inhibitors of metalloproteinases (TIMPs), as well, but in this study, we just aimed to demonstrate the local expression without going into the functionality discourse.

Several limitations of our study have to be considered. Our collection of explanted meshes represents a selection from patients who apparently had a recurrence of an abdominal wall hernia with different intervals after implantation in nonhealthy tissues. Furthermore, only a limited sample size was available, covering three different mesh types with different textile properties (e.g., textile structures, pore sizes). In addition to the many confounders given by the complex staining protocol, it is the location and size of the investigated region that affect the results. Therefore, we used a Euclidean distance map algorithm that created six regional zones adjacent to the mesh fibers. This allowed us to spatially quantify cells and collagen deposits. Our choice of six regional zones, which covered 350 µm from the mesh interface ensured that most of the inflammatory infiltrate around each mesh fiber was included. By relating the total number of cells or positive signal area to the total area of the regional zone, we were able to create a robust, normalized measurement. For cells, the analysis of the staining intensity only in the area of the nuclei cannot exclude positive staining based on some overlapping cytoplasm membrane, but the probability is considerably reduced by sectioning at a thickness of about 2 µm. In addition, we applied a cutoff value of 100 for the mean staining intensity of cells, as described [13]. This allowed us to determine positive cells in an objective and reliable way and provided us with the most confidence for true positive detection.

In addition, as mentioned, macrophage polarization nomenclature is under scrutiny because of overlapping markers and the broad spectrum of macrophage origin and functional distinctions in tissues [26]. Hence, even though CD86 is considered an M1-specific marker, it can be identified in the M2 macrophage subset: M2b. Therefore, although we label these cells M1 for simplicity, the reality is more complex, and these could also be M2b or other macrophages in the multidimensional spectrum of polarization. Regardless of nomenclature, our findings do not implicate M1 or M2b cells as major components of the mesh implants we examined. In addition, even though we report on two M2 macrophage groups (CD163^+^ or CD206^+^), we acknowledge that these may indicate the same cells, as M2a and M2c cells can express both markers [27], while tumor macrophage studies show approximately half of CD206^+^ macrophages expressed CD163 [28]. Although it may be useful to fully categorize these cells with MALDI proteomics approaches for example, this was beyond the scope of our study—which aimed to address which broad category of macrophage (inflamed or wound healing) was related to collagen deposition at the foreign body granuloma.

In conclusion, the findings of the present study suggest that the foreign body reaction to PP meshes is associated with an M2 response that persists even years after implantation and is related to collagen deposition and turnover. Therefore, our findings challenge the idea that a stimulated transformation of macrophages towards the M2 phenotype is a solution to ameliorate the long-term foreign body reaction [11, 14, 29]. Future studies are required to assess whether these M2 macrophages are responsible for excessive fibrosis with all its clinical side effects, and whether attenuating this response is able to reduce complications after abdominal wall hernia repair.

## Electronic supplementary material

Below is the link to the electronic supplementary material.

Supplementary file1 (PDF 841 kb)

## Data Availability

The raw data required to reproduce these findings are available on request from the authors.
