# Supplementary material for: Tissue remodeling macrophages morphologically dominate at the interface of polypropylene surgical meshes in the human abdomen
Source: Hernia. 2020 Oct 8;24(6):1175–89. doi: 10.1007/s10029-020-02315-2 (PMC7701072; doi:10.1007/s10029-020-02315-2)
Supplement: Supplementary file 1 — Supplementary file1 (PDF 841 kb) [file 10029_2020_2315_MOESM1_ESM.pdf]

Tissue remodeling macrophages morphologically dominate at the interface of polypropylene surgical meshes in the human abdomen

Journal HERNIA

Authors *Axel Dievernich[1], Pascal Achenbach[2], Luke Davies[3], Uwe Klinge[1]

[1] Department of General, Visceral and Transplant Surgery, RWTH Aachen University Hospital, Aachen, Germany

[2] Institute of Neuropathology, RWTH Aachen University Hospital, Aachen, Germany

[3] Division of Infection and Immunity, Cardiff University, Cardiff, UK

* Corresponding author

A. Dievernich

Department of General, Visceral and Transplant Surgery, RWTH Aachen University Hospital, Pauwelsstraße 30, 52074 Aachen, Germany

e-mail: adievernich@web.de

Control tissues

In healthy human control tissues of tonsil and spleen we always found some cells that stained positive for CD68, CD86, CD163, or CD206, but M1 ($CD68^{+}CD86^{+}$) and M2 macrophages ($CD68^{+}CD163^{+}$ or $CD68^{+}CD206^{+}$) were rare (<2% of all cells) (s-Table 1).

In the spleen, we found $CD68^{+}$ cells in the marginal zone as well as the red and white pulp (s-Fig. 1). In addition, we observed abundant $CD206^{+}$ cells (14.4%) in the marginal zone rarely co-expressing CD68 and as expected $CD163^{+}$ red pulp macrophages (5.8%), 31% of which co-expressed CD68.

Figures

s-Fig. 1 Immunofluorescence labelling of macrophages in human spleen tissue. (a) Labelling of CD68 (FITC, green), CD163 (Cy5, red), and nuclei (DAPI, blue), and (b) of CD68 (FITC, green), CD206 (Cy5, red), and nuclei (DAPI, blue). 31% of the red pulp macrophages ($CD163^{+}$) co-expressed CD68, whereas co-expression with CD206 was rare (<0.1%)

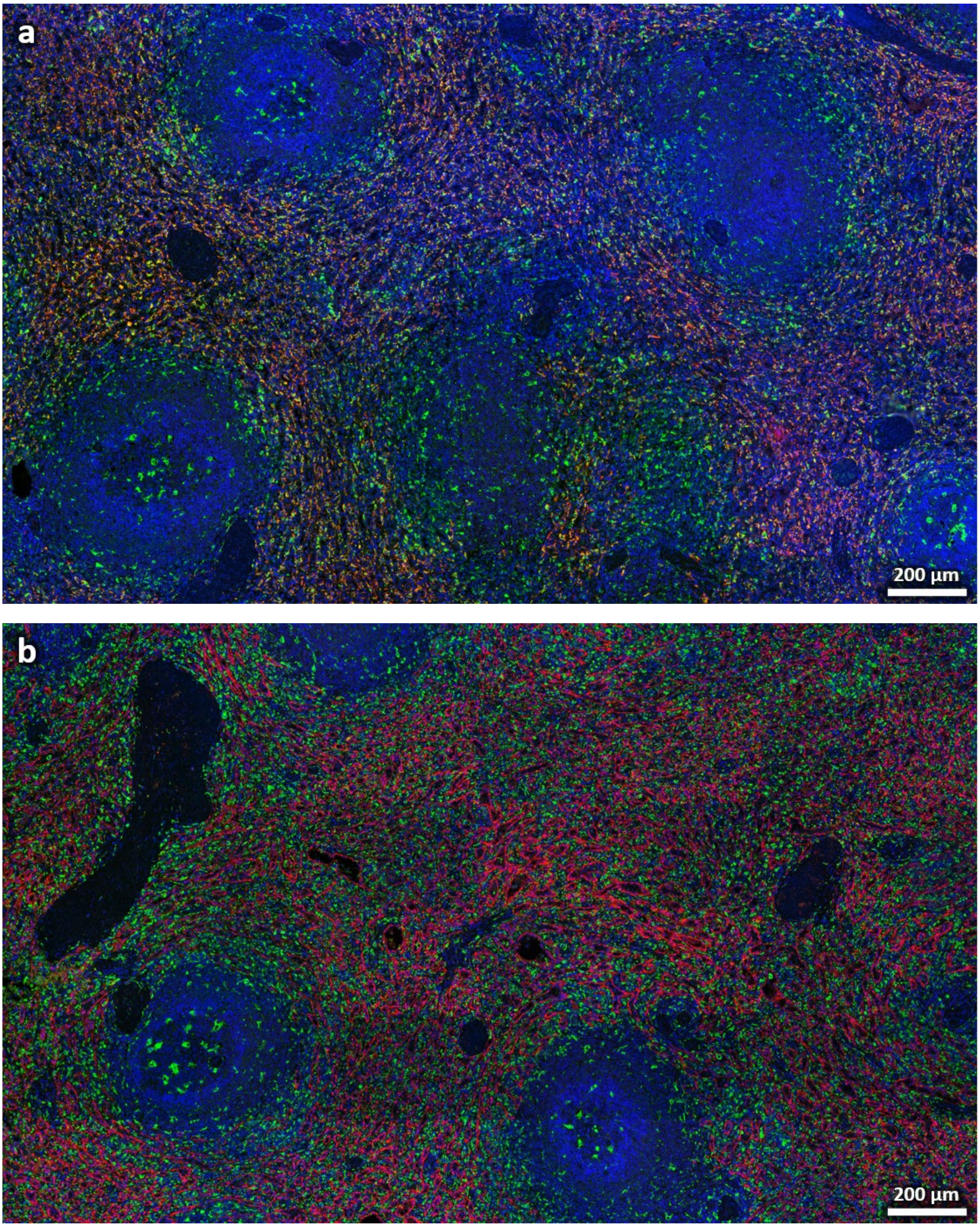

Tables

s-Table 1 Percentages of macrophages in control tissues. Results of the examination of healthy human tonsil and spleen tissues for M1 macrophages ($CD68^+CD86^+$) and M2 macrophages ($CD68^+CD163^+$ or $CD68^+CD206^+$). In each tissue we selected 3 non-overlapping rectangular regions of interest (ROIs) with an area of 2 mm². Cells with a mean staining intensity above 100 were considered "positive". The data are presented as mean (SE).

**Parameter** (n = 3 ROIs; area = 2 mm²)	**Control Tissues**	
	Tonsil	*Spleen*
cell density [cells/mm^2]	16047.7 (241.0)	11343.7 (303.4)
% $CD68^+$ cells	2.4 (0.2)	6.8 (0.5)
% $CD86^+$ cells	0.2 (0.0)	0.3 (0.1)
% $CD68^+CD86^+$ cells	0.0 (0.0)	0.0 (0.0)
% $CD68^+CD86^+/CD68^+$	1.0 (0.3)	0.0 (0.0)
% $CD163^+$ cells	0.1 (0.0)	5.8 (1.2)
% $CD68^+CD163^+$ cells	0.0 (0.0)	1.9 (0.5)
% $CD68^+CD163^+/CD68^+$	0.2 (0.0)	31.1 (5.4)
% $CD206^+$ cells	0.1 (0.1)	14.4 (1.7)
% $CD68^+CD206^+$ cells	0.0 (0.0)	0.1 (0.0)
% $CD68^+CD206^+/CD68^+$	0.2 (0.1)	0.8 (0.2)
